# A data-driven, hyper-realistic method for visualizing individual mental representations of faces

**DOI:** 10.3389/fpsyg.2022.997498

**Published:** 2022-09-28

**Authors:** Daniel N. Albohn, Stefan Uddenberg, Alexander Todorov

**Affiliations:** Booth School of Business, The University of Chicago, Chicago, IL, United States

**Keywords:** StyleGAN, reverse correlation, face judgments, data-driven, face perception

## Abstract

Research in person and face perception has broadly focused on group-level consensus that individuals hold when making judgments of others (e.g., “X type of face looks trustworthy”). However, a growing body of research demonstrates that individual variation is larger than shared, stimulus-level variation for many social trait judgments. Despite this insight, little research to date has focused on building and explaining individual models of face perception. Studies and methodologies that have examined individual models are limited in what visualizations they can reliably produce to either noisy and blurry or computer avatar representations. Methods that produce low-fidelity visual representations inhibit generalizability by being clearly computer manipulated and produced. In the present work, we introduce a novel paradigm to visualize individual models of face judgments by leveraging state-of-the-art computer vision methods. Our proposed method can produce a set of photorealistic face images that correspond to an individual's mental representation of a specific attribute across a variety of attribute intensities. We provide a proof-of-concept study which examines perceived trustworthiness/untrustworthiness and masculinity/femininity. We close with a discussion of future work to substantiate our proposed method.

## Introduction

What types of faces do individuals draw to mind when they think of a “leader”? A “criminal”? A “thinker”? Understanding the content and downstream consequences of such visual stereotypes has been an area of active study within the field of social perception. However, the dominant approach to characterizing judgments derived from faces and facial attributes involves drawing conclusions based on aggregated measures collected from disparate observers. For example, one highly replicable finding is that neutral faces that have more feminine attributes (e.g., lighter skin, rounded jawline) and appear happier are judged as more trustworthy (see, e.g., Oosterhof and Todorov, 2008; Jaeger and Jones, 2021). However, this observation is based on averaged judgments across both faces and raters (i.e., participants). That is, *on average*, any given face with feminine and happy-like features will likely be judged by a given individual as trustworthy. However, not every feminine/smiling face is necessarily seen as trustworthy. Similarly, not every individual agrees on what a “trustworthy” face might look like. What one person may view as “trustworthy” another might view as “gullible” or perhaps “intelligent.” These kinds of idiosyncrasies in face judgment are as numerous as the number of personality traits an individual can judge.

In the present work, we present a rationale for parsing, analyzing, and interpreting both participant-level (i.e., idiosyncratic) and stimulus-level (i.e., shared) contributions to social judgments of faces. First, we review literature that suggests idiosyncratic variance is both pervasive and meaningful in social trait judgments. However, we show that its contribution to social judgments depends on the type of judgment. Specifically, we use two types of social judgments: first-order facial judgments of masculinity and femininity and second-order, more complex judgments of trustworthiness (which rely on integrating many different lower-level perceived attributes in faces). While strict delineation between first- and second-order judgments is difficult to quantify, in the present work we define first-order judgments as those that have clear phenotypic qualities, such as sexually dimorphic features (e.g., jaw shape, face roundness), eye size, and overt or incidental resemblance to emotion expressions. These judgments are likely to have greater inter-rater agreement due to the physically observable features. Similarly, we define second-order judgments as more abstract judgments (e.g., trustworthiness) that are influenced by first-order judgments. Second-order judgments typically have lower inter-rater agreement. For example, the perceived second-order judgment of “babyfacedness” has been shown to be influenced by first-order judgments of face roundness, eye size, and incidental facial resemblance to fear expressions (Marsh et al., 2005; Zebrowitz, 2017). Here, we show that judgments of masculinity and femininity more closely align with our definition of first-order judgments, while judgments of trustworthiness align with what we define as second-order judgments.

Second, we review advances in machine learning and computer vision that can aid in capturing both idiosyncratic and shared variance in face judgments. Third, we introduce a novel, data-driven methodology to visualize idiosyncratic models of faces utilizing machine learning. Finally, we end with a proof-of-concept demonstration of our proposed method. We model idiosyncratic representations of perceived masculinity/femininity and trustworthiness of faces. Consistent with the variance component analyses, we find that these representations are much more similar across individuals in the case of masculinity/femininity than in the case of trustworthiness. We conclude with a set of future directions needed to further validate the proposed method.

## Idiosyncratic and shared contributions to face judgments

Intuitively, two or more individuals are likely to disagree to some degree on their opinions about the attractiveness, trustworthiness, or masculinity and femininity of an individual. Indeed, there is a growing literature on individual preferences across diverse domains such as abstract art (Leder et al., 2016; Specker et al., 2020), architecture (Vessel et al., 2018), dancing (Isik and Vessel, 2019), facial beauty (Hönekopp, 2006; Martinez et al., 2020), voices (Lavan et al., 2020), and even technical writing and peer reviews (Jirschitzka et al., 2017). Yet, traditional analyses in person perception aggregate judgments to focus solely on the “shared” contributions of preferences that are similar across all participants. Thus, the majority of past research in face preferences stands in contrast to emerging evidence on the importance of idiosyncratic contributions to preference, taste, and judgments.

Estimating shared and idiosyncratic contributions to social judgments requires calculating variance components at three levels of interest: the stimulus, the participant, and their interaction (Martinez et al., 2020). The stimulus component represents the “shared” variance that is similar across all raters in the sample. For example, if every judge rated all smiling faces as more attractive, this would be reflected in the stimulus component. In other words, the “shared” stimulus attribute of smiling accounts for a certain proportion of the observed variance. On the other hand, the other two components represent participant-level, idiosyncratic contributions to judgments. The participant main effect contributions are idiosyncratic, but are often considered more ambiguous to interpret. For example, Rater A may judge the perceived happiness of two faces as *numerically* different from Rater B, but both raters could still rank order them similarly, resulting in mean differences across participants but identical face rankings. Such a case would be reflected in the participant main effect variance component, but it is unclear whether such differences reflect true idiosyncrasies or simply the fact that different participants interpret the response scale differently. On the other hand, the participant by stimulus interaction component is more straightforward to interpret. This component captures individual ranking preferences for the stimuli. For example, variance at this level will occur if Rater A prefers (or gives a higher perceived attribute rating to) one stimulus over another while Rater B prefers the opposite pairing (for detailed discussion see Hönekopp, 2006; Martinez et al., 2020).

In an early investigation on social judgment idiosyncrasies, Hönekopp (2006) revealed that perceptions of facial attractiveness were explained by *both* individual and shared preferences in taste. Hönekopp first had participants rate the same faces twice on perceived attractiveness one week apart. Next, shared and private (i.e., idiosyncratic) taste in attractiveness was evaluated by determining the proportion of variance explained by the stimulus face image, the rater, and the rater by stimulus interaction. Shared, stimulus-level contributions in taste accounted for approximately 33% of the observed variance while private, participant-level contributions in taste accounted for about 26% to 45% of the observed variance depending on whether the participant main effect variance was taken into account. Hönekopp (2006) determined that both individual- and stimulus-level contributions were important determinants of attractiveness judgments. Other investigations have shown that idiosyncratic variance contributions to attractiveness judgments range from 20 to 40% conservatively (taking into account participant by stimulus interactions only) to well over 50% if the more ambiguous participant main effect variance component is included (Hehman et al., 2017; Martinez et al., 2020).

**Figure 1:**
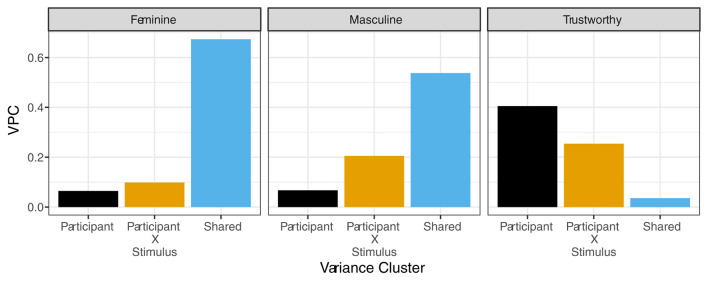
The variance partitioning coefficients (VPC) for feminine, masculine, and trustworthy judgment ratings across important variance clusters. The x-axis represents idiosyncratic (participant and participant by stimulus) and shared variance clusters. The y-axis represents the proportion of observed variance explained by each cluster.

Despite increasing evidence that there are large idiosyncratic contributions to judgments across a variety of domains, the degree to which there is more idiosyncratic variance over shared variance is likely graded within these specific domains. For example, within the domain of facial judgments, low level, first-order judgments that underlie higher level, second-order judgments are likely to have higher agreement. First-order judgments such as those for masculinity, femininity, skin tone, hair color, face shape, among others are likely to have more shared agreement since these attributes tend to be less perceptually ambiguous. In contrast, there is likely to be less agreement (i.e., more idiosyncratic contributions) for second-order judgments, such as those for attractiveness, trustworthiness, and dominance due to highly individualized preferences for these perceived attributes. In our own work, we have found evidence for such effects (Albohn, Martinez and Todorov, *in prep*). We had participants (*N* = 99) judge the femininity, masculinity, or trustworthiness of 120 neutral faces from the Chicago Face Database (Ma et al., 2015). Next, we computed the shared and idiosyncratic variance components following the procedures outlined by Martinez et al. (2020). When we examined the relative proportion of each type of variance, we observed different patterns dependent on whether the judgment was first-order (feminine or masculine) or second-order (trustworthy) as depicted in Figure 1. Specifically, we found that shared variance in judgments of facial masculinity and femininity accounted for approximately 60% of the observed reliable variance (depicted *via* the large blue bars in the left and center panels) but <4% of the reliable shared variance in judgments of trustworthiness (depicted *via* the small blue bar in the right panel). Importantly, this pattern flips when idiosyncratic contributions are examined. Idiosyncratic variance accounted for <20% of the variance for feminine and masculine face judgments (depicted *via* the black and yellow bars in the left and center panels) and around 20–65% of the idiosyncratic variance for trustworthy judgments (depicted *via* the black and yellow bars in the right panel) depending on whether participant main effect variance components are taken into account. This work aligns with previous research that has similarly found that trustworthiness judgments are often more idiosyncratic compared to judgments for other perceived attributes such as gender and race (Hehman et al., 2017).

## Visualization of individual mental representations of faces

There exist myriad data-driven methods for visualizing mental representation of faces (e.g., Gosselin and Schyns, 2001; Mangini and Biederman, 2004; Oosterhof and Todorov, 2008; Schyns et al., 2009; Todorov et al., 2011; Zhan et al., 2021). However, to our knowledge psychophysical reverse correlation that produces noisy or computer generated avatar images is the only approach used for visualizing *individual* representations of faces (Sekuler et al., 2004; Dotsch and Todorov, 2012; Zhan et al., 2021). Typical reverse correlation in social perception overlays base images with random noise to randomly vary features of those base images (Dotsch et al., 2008). Participants then complete a forced-choice task whereby they choose which of two images (overlaid with randomized noise or its inverse) best represents a perceived target attribute (e.g., “Which face looks more “threatening?”). Afterwards, a classification image can be created for each participant by averaging all of the selected images together. In short, reverse correlation can visualize social perceptions of individuals by allowing a meaningful construct to “emerge from the noise.”

Reverse correlation has become extremely popular in social perception since its inception. Indeed, past work has utilized reverse correlation to exemplify group averages of ethnic groups (Dotsch et al., 2008), social groups (Tskhay and Rule, 2015; Lloyd et al., 2020), facial emotions (Albohn et al., 2019; Albohn and Adams, 2020), and social categories such as perceived trustworthiness and dominance (Dotsch and Todorov, 2012). While many studies utilize reverse correlation for visualizing higher-level perceived attributes in faces, existing techniques can be improved considerably by leveraging advances from computer vision that have occurred in the last decade. Potential areas of improvement include (1) creating individual-level (rather than group-level) models of social perception; and (2) increasing classification image quality. We expand on both of these points in greater detail below.

### Individualized models of social perception

Most studies utilizing reverse correlation only collect and report results from classification images averaged together at the group level. Then, these averaged images are rated by other samples of participants to confirm that such group-level classification images appear as intended (e.g., a “dominant” reverse correlation classification image actually appears “dominant” to other raters). However, the focus on characterizing only group-level images has been shown to inflate Type I error rates (Cone et al., 2020). To address this issue, individual classification images can be rated, though studies utilizing this approach typically still only report group means aggregated over all participants in the study and not individual-level effects. While such approaches have revealed important insights into social perception, they reveal little about the idiosyncrasies of social judgments. For example, it is possible that an individual's classification image of a “dominant” face could accurately visualize their mental representation of perceived facial “dominance,” yet simultaneously fail to correspond to a group-level consensus representation of perceived “dominance.” In such cases, the individual's visualized mental representation would likely either be discarded as an outlier or ignored.

### Improved image quality

As noted earlier, the reverse correlation paradigm is, by its very nature, noisy. Such experiments require the application of carefully calibrated image-based noise to some base image in order to generate the distinct stimuli participants must choose between on each trial. Consequently, the resultant classification images — created by averaging across participant-selected noise patterns — are low fidelity. The images look noisy, pixelated, and blurry, or altogether computer-generated. Early visual reverse correlation techniques utilized stimuli that consisted solely of randomized noise (e.g., Gosselin and Schyns, 2003). Such noise-only methods typically required each participant to complete thousands of trials in order for meaningful data to emerge. In light of this difficulty, later iterations of visual reverse correlation made use of a blurred base image, on top of which the noise was applied (Mangini and Biederman, 2004; Dotsch et al., 2008). This helps guide visual classification and reduces the number of required trials by an order of magnitude — from thousands to several hundred. However, the resultant classification images can only be as clear as the images used in their creation. Even when randomized noise is superimposed over a high-fidelity base image, the output classification image is still necessarily noisy and blurry in appearance. Classification images are *prima facie* computer-manipulated and participants are unlikely to believe the images represent “real” faces, although human judges can still consensually discern gross anatomical features (e.g., mouth, eyes, hair, brows, etc), some social categories (e.g., race, gender/sex), and face luminance from noisy classification images.

### A promising way forward

Addressing the existing limitations of psychophysical reverse correlation would allow for several important avenues of exploration in future research. First, a reverse correlation approach that yields photorealistic face images would have high face validity. This is because participants would likely be unable to tell that the images they are categorizing (or rating) are synthetic, leading to greater ecological validity. For example, prior work has demonstrated that judging the trustworthiness of computer-generated faces (as compared to real faces) results in weaker observed effects (Balas and Pacella, 2017). Relatedly, computer-generated and artificial faces have been shown to be more difficult to process and remember compared to real faces (Balas and Pacella, 2017; Gaither et al., 2019). Therefore, it is likely that there is less cognitive burden for rating photorealistic face images compared to pixelated images or avatars.

Second, a higher-fidelity approach to reverse correlation would allow for easier and more effective integration with other computational methods in the social sciences. Traditional approaches produce noisy or low-fidelity classification images which are not discernible by pipelines that use computer vision to automatically calculate structural and surface properties of the face in addition to other metrics that may be of interest to researchers. In contrast, real face images can often be substituted one-for-one with photorealistic computer generated face images in algorithms that can automatically detect or estimate facial landmarks, self-identified demographic properties (e.g., race, age, sex/gender), and emotional expression, among other such perceived attributes. In summary, more realistic classification images are ideal not only for human observers to make high-quality judgments, but also for algorithms that can aid in extracting and interpreting lower-level face metrics.

## Advances in machine learning applications

One of the most important recent advances to computer vision has been the generation of photorealistic synthetic images. Generative adversarial networks (GAN) accomplish this by pitting two machine learning models in “competition” against each other with the goal of creating better and better output. A generator model produces synthetic output data (often an image) in an attempt to “fool” a separate discriminator model simultaneously trained to discern “real” from “synthetic” data (Goodfellow et al., 2014). Generative adversarial networks have been used to create a variety of image classes, ranging from faces to house facades, cars, and animals, among many others. A generative model learns to produce new images by dynamically updating its output based on whether or not the discriminator model can tell whether its output is a real image or a generated image. Similarly, the discriminator model dynamically improves its performance using the feedback it receives from the generator model.

One GAN that has received considerable attention and research is StyleGAN, a machine learning model capable of producing different types of images at high resolution that are nearly indistinguishable from real world photos (Karras et al., 2018, 2020, 2021). Of particular interest here, StyleGAN has been able to generate human faces with incredible fidelity and precision, mimicking real-world face photographs while also being able to construct new face images of non-existent individuals.

In addition to creating high resolution output, StyleGAN has also proven to be reliable in manipulating faces along a number of dimensions of psychological interest. For example, researchers were able to identify where in the StyleGAN latent space demographic attributes such as age and sex/gender exist. Identifying latent directions for such attributes then allows for any images generated to be moved along those directions and thereby manipulated along age (young to old) or sex/gender (perceived male to perceived female), or both (Shen et al., 2020).

**Figure 2:**
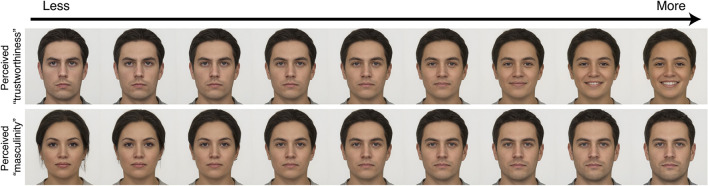
An average of 2,484 neutral faces projected into the StyleGAN2 latent space and transformed along dimensions of perceived “trustworthiness” and “masculinity.” These dimensions were modeled using average ratings for 1,004 faces each, collected from a general online participant population (Peterson et al., 2022). The faces along each step of the group-level continua are highly realistic and evoke vivid impressions of the transformed perceived attribute.

There is nothing limiting the discovery of latent directions within the latent space to such (first-order) demographic features. For example, one recent paper applied modeling techniques previously used for characterizing representations of psychological perceived attributes in 3D computer-generated faces (e.g., Oosterhof and Todorov, 2008) to the StyleGAN2 latent space (Peterson et al., 2022). By collecting over 1 million judgments of 1,004 synthetic faces from over 4,150 participants, the researchers visualized 34 perceived attribute dimensions. These dimensions included such first-order judged dimensions as perceived “masculinity,” as well as second-order judged dimensions such as perceived “trustworthiness.” These perceived dimensions in particular were able to be modeled with great fidelity, owing to the high inter-rater reliability of participants' judgments. The results are best appreciated with a demonstration: Figure 2 depicts transformations of these two perceived dimensions applied to an averaged neutral face (itself the average of almost 2,500 neutral faces collected from various extant stimulus sets, and described in more detail in the Methods section below).

To our knowledge, this approach represents the state of the art for group-level modeling of social perception in faces (Peterson et al., 2022). However, this set of studies was explicitly designed to characterize the mental representations of faces in the general (online) population and could not model the idiosyncratic representations of individual participants. In principle, the same approach could capture such individualized models simply *via* “scaling up” data collection — by asking a given participant to contribute over a thousand judgments per perceived attribute of interest, it should be possible to characterize their idiosyncratic visual representations. In practice, however, this would prove to be time consuming to the participant, costly to the experimenter, and potentially self-defeating by nature of its greater scale; the approach would likely yield lower and lower quality data as the experiment progresses. Ideally, modeling such perceived dimensions or attributes in face space would not require thousands of trials from each participant. Here, we have developed one promising approach to do so that requires only a few hundred trials, and combines advances in GAN-based image generation with the previously discussed psychophysical reverse correlation techniques.

While our work relies on a particular type of GAN for the generation and transformation of faces, the field of machine learning continues to rapidly advance and new methods emerge regularly. Recently, research groups from companies such as OpenAI (Ramesh et al., 2022) and Google (Saharia et al., 2022) have created especially powerful generative diffusion models (DALL-E 2 and Imagen, respectively) *via* a technique known as Contrastive-Language-Image-Pretraining (CLIP; Radford et al., 2021). Among other things, these models allow for the creation of arbitrary images from text prompts, which can themselves be quite descriptive (e.g., “a racoon detective in New York City wearing a trench coat under a street light.”). In addition, it is possible to quickly generate many variants of a given image, or to edit the content of an image by applying a mask to an area of the image and asking for a desired change with further text (e.g., “add a bed” to a masked area of a scene, which will then be filled with a bed). The availability of such models to the general public is currently limited for various reasons (including concerns of potential abuse by unethical actors) but may prove enormously useful to the broader psychological research community, both for stimulus generation and analysis/exploration of participant data. It is worth noting that although the human faces generated by these types of models are currently not as realistic as those generated by the likes of StyleGAN, it seems likely that they will close the gap in the months to come; future work should explore the utility of such techniques, as they may allow for even more diverse and naturalistic stimuli.

## Summary and overview

To summarize the arguments above, typical research in person perception analyzes mental constructs of interest by aggregating over both stimuli and individual participant raters. As such, reported results are only interpretable at the highest group level (e.g., on average a given face image is viewed by a typical participant as “trustworthy”). While aggregated results are informative, emerging research suggests that within person perception a large portion of the variance is attributable to idiosyncratic differences in raters, particularly for complex perceived attributes such as trustworthiness.

Despite both increased attention to idiosyncratic contributions to social perceptions and advances in technology, there is still no reliable, high-fidelity method for visually inspecting individual mental representations of perceived personality attributes. In the present work, we introduce a novel, data-driven method for visualizing hyper-realistic mental representations of social judgments of perceived facial attributes. To accomplish this, we leverage state-of-the-art machine learning models to generate manipulable, highly realistic faces.

We first formally introduce our proposed methodology and then apply it to a small proof-of-concept study. Our main goal is to show that our procedure can visually capture individual social judgments in a predictable manner. For our initial investigation, we utilized both a first-order social judgment (“feminine/masculine”) and a second-order social judgment (“trustworthy”). The two judgments were selected intentionally to visualize attributes that should theoretically vary with respect to the shared and idiosyncratic contributions to judgments (see Figure 1). More specifically, while we predicted that all individual mental representations would be idiosyncratic to some degree, we also predicted that first-order social judgments (i.e., feminine/masculine) would vary less across individuals compared with second-order social judgments (i.e., trustworthy). This prediction aligns with previous work on shared and idiosyncratic contributions to social judgments.

## Initial proof-of-concept study

### Methods

The proposed methodology for our hyper-realistic visualization procedure loosely follows that of a typical psychophysical reverse correlation procedure (Dotsch and Todorov, 2012). However, there are several major differences whereby we leverage state-of-the-art technical innovations. Specifically, our methodological procedure consists of four steps: (1) image inversion; (2) stimulus creation; (3) stimulus selection (by participants); and (4) stimulus analysis (i.e., classification image creation). In what follows, we first briefly introduce each of our methodological steps and then detail the results from a proof-of-concept investigation using our method.

#### Step 1: Image inversion

The first step consists of inverting a set of faces into the StyleGAN2 latent space. In short, GAN inversion is a process whereby a real face image is reconstructed (located) within a pre-trained GAN latent space (Xia et al., 2022). A successful inversion results in an image that is photorealistic, similar in appearance to the original image, and editable (i.e., maintains the same characteristics of the GAN latent space into which it was inverted so that attributes present in the latent space can be applied to the inverted image). Inverting an image results in a 18×512 matrix of numeric values that represents that face in the StyleGAN2 latent space.

We inverted 2,484 neutral faces from various available databases into the StyleGAN2 latent space using a modified VGG encoder adapted by Peterson et al. (2022). The neutral faces were taken from several face databases: the Chicago Face Database (Ma et al., 2015), FACES (Ebner et al., 2010), NIMSTIM (Tottenham et al., 2009), RAFD (Langner et al., 2010), Face Database (Minear and Park, 2004), Face Research Set London (DeBruine and Jones, 2017), FERET (Phillips et al., 2000), and RADIATE (Conley et al., 2018) image sets, as well as a number of internal face resources. We focused on inverting real neutral face images because the original StyleGAN2 latent space is oversaturated with smiling faces due to the nature of the original training data (Karras et al., 2020). An overrepresentation of smiling faces (or any other type of face/attribute) is undesirable for obtaining an accurate classification image (i.e., individual face prototype or representation). In our tests, an oversampling of smiling faces in the image pool shown to participants resulted in classification images that also overrepresented “smiley” attributes.

#### Step 2: Stimulus creation

In a typical reverse correlation study, stimuli are created by overlaying random sinusoidal noise over a standardized, singular base image. Much like the original reverse correlation procedure, we sought to create stimuli for our experiment by randomly generating random neutral faces from the GAN latent space and adding a small amount of Gaussian noise. To generate random, unique neutral faces from the latent space, we first averaged together the latents of a subset of 10 randomly selected faces from the 2,484 faces inverted into the model latent space in the previous step. Next, we added a small amount of random Gaussian noise to the averaged latent to further differentiate it from the pool of inverted faces. This two-step process was repeated for each stimulus generated.

**Figure 3:**
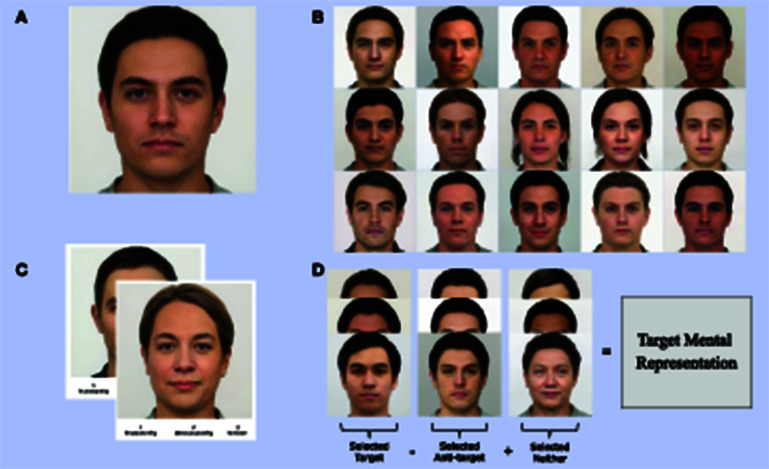
Overview of proposed method. **(A)** Depicts the average of 2,484 neutral faces inverted into the StyleGAN2 latent space. **(B)** Provides example neutral face images created by adding random noise to inverted neutral face images. **(C)** Displays example trials participants saw during “stimulus selection” (Step 3). **(D)** Provides an overview of calculating the average directional vector of a target trait applied to an average face to create a target mental representation.

We generated 300 neutral face stimuli utilizing the procedure outlined above. We sampled noise from a Gaussian distribution with parameters μ = 0 and σ = 0.4 to be added to each generated average image. Examples of the generated stimuli with these parameters can be seen in Figure 3B.

#### Step 3: Stimulus selection

Our stimulus selection procedure involves displaying each image sequentially to participants and asking them to categorize the face based on a specific set of attributes designated by the researcher. In contrast to a standard reverse correlation procedure, which typically uses a two-alternative forced-choice design (i.e., selecting between two images), we propose utilizing a design with three potential categorizations for a single image–that is, a single image is displayed to the participant along with three response options. In our task, each of the categories is as follows: (1) the attribute of interest (e.g., perceived “trustworthy”), (2) the conceptual opposite of this attribute (e.g., perceived “untrustworthy”), and (3) “neutral” (or “neither”). For example, if a researcher is interested in visualizing an individual's mental representation of perceived “masculinity,” participants would be asked to select whether they think each face appears “masculine,” “feminine,” or “neither.” The rationale for including a “neutral” or “neither” category is to obtain an unbiased, individual starting point within the latent space for each participant. That is, much like the target categories, what one individual categorizes as “neither” is likely to differ from one participant to the next. The participantHeading 3,H3,APA Level 2s selections are binned into each of the three categories and used for analysis in the next step.

The current experiment had two sets of judgments: “trustworthy/untrustworthy/neither” and “masculine/feminine/neither.” Participants were assigned to one of the conditions and tasked with categorizing each face stimulus into one of the three categories. Every participant saw the same 300 faces generated in the previous step, though presentation order was randomized between participants.

#### Step 4: Stimulus analysis

Stimulus analysis involves matrix arithmetic on the latents of the selected images for each participant. First, the image latents for each selected category are averaged together. Second, the latent matrix of the non-target category is subtracted from the latent matrix of the target category. This process isolates the unique features attributable to the perceived target attribute in the latent space. For example, subtracting the average “untrustworthy” latent matrix from the average “trustworthy” latent matrix yields a new latent matrix that represents the qualities unique to perceived trustworthiness for a given participant. The result of this operation represents a directional vector in the latent space that can be used to sample the mental representation of the desired perceived attribute at varying levels of intensity. This is accomplished by adding the directional vector to the averaged latent matrix of the “neither” selections, which represents a starting point in the StyleGAN2 latent space for estimating an individual's mental model for a particular trait. Consequently, multiplying the directional vector matrix by a constant before adding it to the averaged “neither” latent matrix produces visualizable mental representations for the trait at different levels of intensity.

More formally, *A* ∈ ℝ^*m x n*^ is an 18 × 512 dimensional matrix that represents the averaged latents for the faces selected to represent a target trait. Similarly, *B* ∈ ℝ^*m x n*^ is a 18 × 512 matrix of the averaged latents for the non-target or non-selected faces. The directional vector matrix, **A**, for a particular subject, *i*, can be computed as,


$$\begin{array}{l} A_{i}\;=\;A_{i}-B_{i} \end{array}$$

This directional matrix can be applied to the averaged neither latent matrix, *N* ∈ ℝ^**m** **x** **n**^, to compute a starting point of an individual's mental representation of the target trait,


$$\begin{array}{l} M_{i}=N_{i}+A_{i} \end{array}$$

Finally, when the directional vector matrix is multiplied by a constant, *C*, the mental representation image, *M*_*ic*_, can be estimated at varying intensities,


$$\begin{array}{l} M_{ic}=N_{i}+A_{i}\;C \end{array}$$

The extrapolated mental representations exemplify the individual's internal prototypes for the particular trait measured at various levels of intensity.

We computed individual classification images for each of our participants following the procedure outlined above. If participants did not categorize any faces as “neither,” a random sample of 20 faces were drawn from the pool of 300 faces and averaged together as a proxy starting point in the latent space. Experiments utilizing different random subsets of images did not meaningfully change the visual results of the output images.

### Participants

Eleven participants (*M*_*age*_ = 36, *SD*_*age*_ = 5.24) were recruited to complete the study *via* CloudResearch. Participants self-identified as the following: 6 women, 5 men; 8 White, 1 Black, 1 Asian, and 1 Middle Eastern or North African. Six participants were assigned to the “trustworthy/untrustworthy” condition and five participants to the “masculine/feminine” condition. One participant categorized all images as “trustworthy” and was thus not included in our analyses. Two participants did not categorize any face as “neither,” though “neither” selections were low across both conditions (*M*_*masculine*/*feminine*_ = 6.8%, *M*_*trustworthy*_ = 10.6%). This study was reviewed and approved by the Institutional Review Board at the authors' institution. All participants agreed to take part in this study and were compensated $5 for completing the study.

### Participant procedure

Participants were instructed that they would be shown several hundred face images and would be asked to categorize each face into one of three categories depending on which condition they were assigned. Participants were also instructed to take a moment to imagine what a trustworthy/masculine and untrustworthy/feminine face looked like to them (depending on the condition to which they were assigned).

Next, participants completed the main portion of the experiment whereby they categorized each face. During each of the 300 trials, participants were presented with a face toward the center of the screen along with each of the three categories listed beneath the face. Participants were instructed to use the number keys to make their selection (1 = “trustworthy”/“masculine,” 2 = “untrustworthy”/“feminine,” and 3 = “neither,” as was appropriate for their assigned condition). After completing this portion of the experiment, participants answered a basic demographic questionnaire (e.g., listing their age, race, and gender) and were debriefed.

## Results and discussion

**Figure 4:**
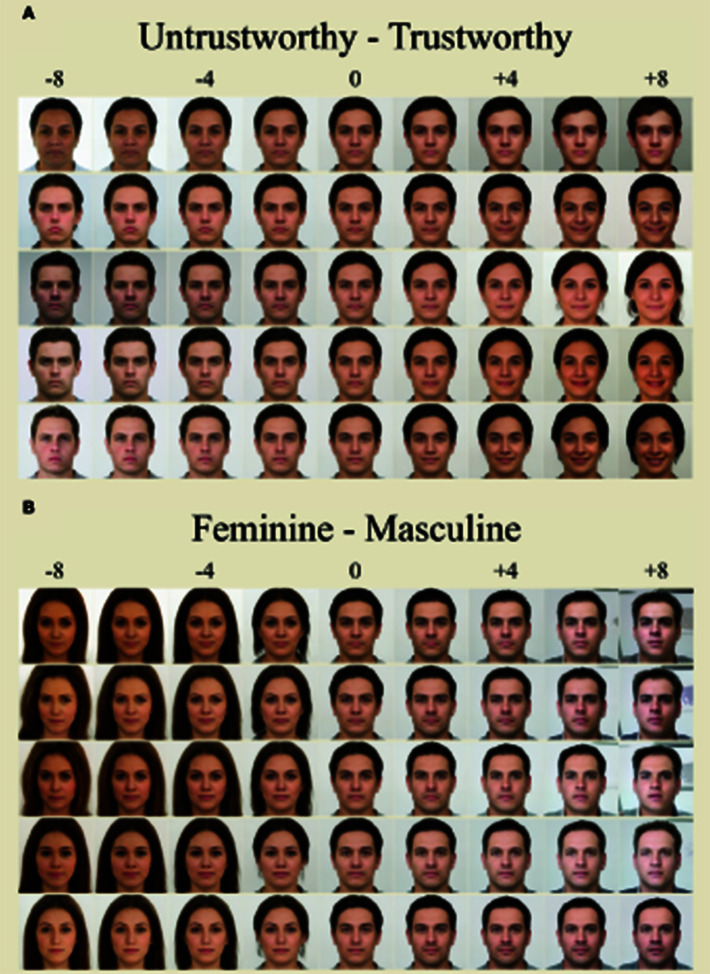
**(A)** Visualizes the mental representations for each participant in the “trustworthy” condition. **(B)** Visualizes the mental representations for each participant in the “masculine” condition. Each row represents a single participant. Each column visualizes mental representations when the directional vector is applied to the average of all faces that were selected as “neither” (0) multiplied by specific constants (−8 to +8).

Figure 4 presents the results from each of the ten participants in our study at several levels of target trait intensity. Individual mental representations were computed following the steps outlined in the Method section. Through experimentation we determined that the directional vector within the latent space could be extrapolated with constants between −8 and +8 without degrading the clarity of the internal face portion of the image or collapsing the latent space (i.e., creating an unrecognizable image). Visual inspection of the produced images suggests that changes between models become readily apparent beginning at +/−4 from the center (or averaged “neither”) starting image. Hence, we suggest that internal prototypes should be examined at values +/−4 or greater to secure an adequate visual representation for each individual mental representation.

Visual inspection of these results confirms the face validity of our proposed procedure. Specifically, individuals assigned to the “trustworthy/untrustworthy” condition yielded visualized mental models of faces that appeared happier and more feminine for trustworthy (positive constant values) as well as angrier/stoic and more masculine for untrustworthy (negative constant values). Participants assigned to the “masculine/feminine” condition yielded visualized mental models of faces that appeared more masculine (positive constant values) as well as more feminine (negative constant values).

While interpreting results from such a small sample of participants warrants caution, there are a few additional aspects of the current results that deserve discussion. For one, it is interesting that prototypical, toothy smiles still occurred at the extremes of perceived trustworthiness despite the neutral appearance of all the stimuli that participants categorized. This pattern of results suggests that overt, robust physical features are still able to be derived from faces that only incidentally resemble them.

Most importantly in terms of idiosyncratic contributions to judgments, participants' mental representations of extremely trustworthy faces varied dramatically in physical appearance (e.g., presenting as both “male” and “female” and at differing ages), underscoring the idiosyncrasies of how each participant imagines “trustworthy” in their mind's eye. Conversely, the mental representations of “masculine” and “feminine” at the extremes appear quite similar in physical appearance (e.g., all appearing feminine with round faces and long hair), underscoring agreed upon characteristics for these judgments.

Critically, and in line with our hypotheses, average correlations among the trustworthy/untrustworthy latents [*r*_−8_(8)= 0.53, *r*_8_(8)= 0.54] were lower than the average correlations among the masculine/feminine latents [*r*_−8_(8) = 0.82, *r*_8_(8) = 0.84] at both extremes. Lower correlations among participants in the “trustworthy/untrustworthy” condition suggest that they had more idiosyncratic mental representations for what they considered trustworthy compared to participants in the “masculine/feminine” condition. Similarly, higher correlations among participants in the “masculine/feminine” condition suggest that they had more shared representations of what they considered masculine or feminine.

## Discussion

In the present work we highlight the importance of understanding both shared and idiosyncratic contributions to the variance of social judgements. That is, how much of the variance in social judgment ratings can be explained by stimulus-level features (i.e., shared) vs. participant-level preferences (i.e., idiosyncratic). Emerging research suggests that both levels explain an important amount of variance, though the proportion of variance explained by each level appears to be influenced by the type of judgment. For example, shared contributions to judgements of first-order, lower-level judgments such as perceived “masculinity” or “femininity” appear to be larger than idiosyncratic contributions. On the other hand, second-order, higher-level judgments such as perceived “trustworthiness” or “attractiveness” seem to be better explained by idiosyncratic contributions.

While emerging work has demonstrated the importance of understanding contributions to judgments at multiple levels of interest, there has yet to be a method for visually capturing high-fidelity individual mental representations. Here, we attempt to fill this gap by introducing a novel, data-driven, and hyper-realistic method for visualizing individual mental representations of perceived attributes inferred from (or ascribed to) faces. We leverage current machine learning technology to create a set of photorealistic face stimuli, each of which is manipulable along a specific directional vector of interest. For example, our pipeline can compute directional vectors of a perceived attribute, such as perceived “trustworthiness,” at the participant level, which can then be applied to an averaged base face to visualize how an individual represents prototypical “trustworthy” (and anti-trustworthy) faces in their mind's eye.

We conducted a proof-of-concept study utilizing our proposed method whereby we had participants create directional vectors (i.e., mental representations) of “un/trustworthy” and “masculine/feminine” faces. Inspection of our results confirms the face validity of our procedure. Specifically, those assigned to create “trustworthy” mental representations produced face images that appeared happier, younger, and more feminine. Likewise, those assigned to the “masculine/feminine” condition produced face images with sexually dimorphic characteristics (see Figure 4). More importantly, and in line with our predictions, there was much more agreement in the images produced for feminine/masculine-looking faces (a first-order judgment) compared to un/trustworthy-looking faces (a second-order judgment). Such effects were expected given previous work on differential variance contributions to specific types of social judgments.

While correlations between feminine/masculine latents were higher than those between trustworthy/untrustworthy latents, it should be noted that the correlations between trustworthy/untrustworthy latents were still relatively high (*r* = 0.5). These moderate correlations may be due to a number of causes, including “shared” characteristics among created images (e.g., smiling), the entanglement of StyleGAN2 latent space, or the relatively low sample size. One potential cause for moderate correlations among the trustworthy/untrustworthy latents is that many of the trustworthy/untrustworthy images share common features, such as frowning or smiling, which would likely be reflected in a similar pattern among the image latents. Similarly, the StyleGAN2 latent space is known to be entangled, i.e., each latent alters more than one visual attribute as its value changes (Wu et al., 2021). The entangled latent space may result in moderately correlated latents even when they are sampled at random. Finally, we may have observed moderate correlations between the trustworthy/untrustworthy latents due to our sample size. It is likely that all three factors are influencing the relationship between the created image latents and future work should aim at detailing how the latents are related and when highly correlated patterns emerge.

In sum, our proposed pipeline for producing mental representations of social judgments of faces produces high quality, photorealistic visualizations of prototypes at the individual level. Our methodology advances how individual mental representations can be visualized in several important ways. First, our procedure allows for researchers to estimate individual prototypes at various levels of intensity. Our technique allows us to take any number of categorizations and build a vector to move through the latent space in a given direction that represents that individual's internal prototype.

Second, our procedure produces high-fidelity images. The resultant images are photo-realistic and indistinguishable in most cases from non-computer generated images. These images can then be rated by human participants or passed to additional machine learning applications that measure facial attributes or metrics. A method that results in a face image that is indistinguishable from a real face allows for participants to provide an unbiased estimate of the stimulus. Similarly, a face that appears realistic can be interpreted by other machine learning algorithms which would allow additional benefits afforded by them, such as facial landmarks estimation as well as face identification, sex/gender, ethnicity, age, emotion, texture, and color estimation. These additional results would not be possible with lower resolution images.

Finally, higher resolution/fidelity images allow for researchers to identify more subtle differences that can occur across social judgments at the individual level. For example, the images created through our procedure in the anti-masculine (feminine) condition resulted in prototypes that globally looked similar but still differed in minor ways such as hair style, gaze, and mouth curvature. Such differences would likely be indistinguishable when the resultant image is of lower resolution.

### Future work

While our preliminary study provides compelling proof-of-concept visualizations, it is important to follow up this work with additional research to confirm the validity of our methodology and determine any boundary conditions that might exist. We identify four important next steps that we plan to undertake to confirm our mental representation pipeline: (1) validate created mental representations by both the creator and naive raters, (2) replicate our results with more participants across a more diverse set of target attributes and judgments; (3) determine the importance of the underlying distribution of faces used to create stimuli; (4) determine the influence of the number of trials on the outcome image.

A critical and missing step in our pipeline is the validation of the individual models. Following the logic of previous validation studies of consensus judgments (Todorov et al., 2013; Todorov and Oh, 2021), participants' judgments should be more sensitive to differences between faces manipulated by their own model than differences between faces manipulated by models of other participants. Specifically, if the intensity of the perceived attribute is manipulated at multiple levels, the slopes of participants' judgments should be steeper for faces manipulated by their own model than by models of other participants. However, we would expect the magnitude of this effect to be attenuated if the judgment of interest is first-order and thus more likely to have higher inter-individual agreement. Further, validating the image transformations *via* ratings from a group of naive participants would also provide insight into whether the generated mental representation images are consensually interpreted as the judgment of interest. In summary, validation by both the classification image creator and other raters is an important validation step for fully understanding the unique contributions of both idiosyncratic and shared variance in face judgments for this proposed methodology.

Second, it is important to establish that our methodology provides consistent and replicable results across a diverse set of target perceived attributes. In our pilot study we provided evidence that our procedure works for both first order and second order judgments of perceived attributes. However, a more diverse portfolio of social judgments that can be visually derived from our pipeline would only further confirm its validity. While we only tested two social judgments and a handful of participants, the results were visually striking. As such, we are confident that studies with additional participants and attributes will provide equally satisfying results.

Third, it is important to understand the effect of the underlying pool of real face images used to provide a foundation for the generated stimuli. In the current pilot we were not concerned with a balanced underlying pool of faces and instead opted to secure a pool of as many high resolution and standardized neutral faces as possible. Therefore, the underlying distribution of faces used to create our experimental stimuli were unbalanced in terms of race, sex/gender, age, and ethnicity. While the social judgments we selected to examine in our pilot study are less likely to be influenced by such imbalances, other target categories that can be examined through our procedure will likely need stimuli drawn from an underlying balanced face distribution. For example, if a researcher is interested in determining whether perceived emotion differs between mental representations of specific ethnic categories, the underlying distribution of faces that the stimuli are created from needs to be balanced in terms of the target ethnicities. A balanced underlying face set would allow for experimental stimuli to be drawn from (or around) an ethnically-ambiguous base face and reduce any unintentional bias that might result from an unbalanced stimulus set.

Finally, the number of trials used in our procedure needs to be experimentally examined to determine a minimal number of trials to produce optimal images. Based on previous work utilizing reverse correlation in social psychology, we determined that our pilot study should use 300 experimental trials. While 300 trials produced visually appealing results, the number of trials could also be increased or decreased to achieve equal or better results. If the number of experimental trials could be reduced while still producing comparable visual results, it would reduce experiment duration and help reduce participant fatigue. Similarly, if increasing the number of trials could increase the final mental prototype image fidelity, it would produce higher quality results. Either way, understanding the optimal number of experimental trials is critical for operationalizing a final procedure for our methodology.

## Conclusion

It is unlikely that two individuals will judge the same person identically on how trustworthy, attractive, or dominant they appear. Even if these two individuals did agree on a numeric value, it is even more unlikely that they would agree upon the reasons why they arrived at such conclusions. The only way to truly understand what physical features in a face individuals use to inform their impressions is to visually capture what they imagine for any given social judgment prototype. Until now, such possibilities were limited to low-fidelity, pixelated, avatar representations of faces or self-reports. Now, by leveraging state-of-the-art computer vision, we have provided a methodology for visualizing high-fidelity mental representations of social attributes inferred from faces at the individual participant level. Our work represents a critical advance in understanding how social judgements are formed and how they can dramatically differ or remain consistent from individual to individual.

## Data availability statement

The original contributions presented in the study are included in the article/supplementary material, further inquiries can be directed to the corresponding author.

## Ethics statement

The studies involving human participants were reviewed and approved by the University of Chicago Institutional Review Board. The patients/participants provided their written informed consent to participate in this study.

## Author contributions

DA, SU, and AT contributed to the conception and design of the methods and studies. DA conducted the pilot studies and performed statistical analyses. SU contributed to the pilot study design and infrastructure. DA and SU wrote the first draft of the manuscript. All authors revised, read, and approved the submitted version of the manuscript.

## Funding

This work was supported by the Richard N. Rosett Faculty Fellowship at the University of Chicago Booth School of Business.

## Conflict of interest

The authors declare that the research was conducted in the absence of any commercial or financial relationships that could be construed as a potential conflict of interest.

## Publisher's note

All claims expressed in this article are solely those of the authors and do not necessarily represent those of their affiliated organizations, or those of the publisher, the editors and the reviewers. Any product that may be evaluated in this article, or claim that may be made by its manufacturer, is not guaranteed or endorsed by the publisher.
